# Downregulation of long non-coding RNA LINC00460 inhibits the proliferation, migration and invasion, and promotes apoptosis of pancreatic cancer cells via modulation of the miR-320b/ARF1 axis

**DOI:** 10.1080/21655979.2020.1863035

**Published:** 2020-12-21

**Authors:** Jian Cheng, Yanghui Lou, Kai Jiang

**Affiliations:** a Department of Hepatobiliary, Pancreatic and Minimally Invasive Surgery, Zhejiang Provincial People’s Hospital, People’s Hospital of Hangzhou Medical College, Hangzhou City, Zhejiang Province, PR China; b Department of Anesthesiology, Yiwu Maternity and Children Hospital, Yiwu City, Zhejiang Province, PR China

**Keywords:** LINC00460, Pancreatic adenocarcinoma (PAAD), miR-320b, ARF1, proliferation, migration

## Abstract

Pancreatic adenocarcinoma (PAAD) ranks among the most lethal cancers worldwide with high mortality. A marked increase in the level of long non-coding RNA LINC00460 was reported in PAAD patients, in comparison with the healthy controls. However, the underlying mechanisms of the above phenomenon are not yet well understood. Hence, the present study was designed to investigate the molecular mechanism underlying the role of LINC00460 in proliferation, migration and invasion of pancreatic cancer (PC) cells. It was found in our study that LINC00460 knockdown inhibited SW1990 cell proliferation, migration and invasion and promoted its apoptosis. Moreover, miR-320b was targeted straight and its expression was downregulated by LINC00460, whose knockdown led to a reduction in ARF1 expression. Interestingly, miR-320b downregulation partly reversed the effect of LINC00460 knockdown on the proliferation, migration, invasion and apoptosis of SW1990 cells, as well as ARF1expression. In conclusion, LINC00460 knockdown inhibited the proliferation, migration and invasion, and promotes the apoptosis of SW1990 cells via modulation of the miR-320b/ARF1 axis. Thus, LINC00460 can be perceived as a promising target in the treatment of PAAD.

## Graphical Abstract

**Figure:**
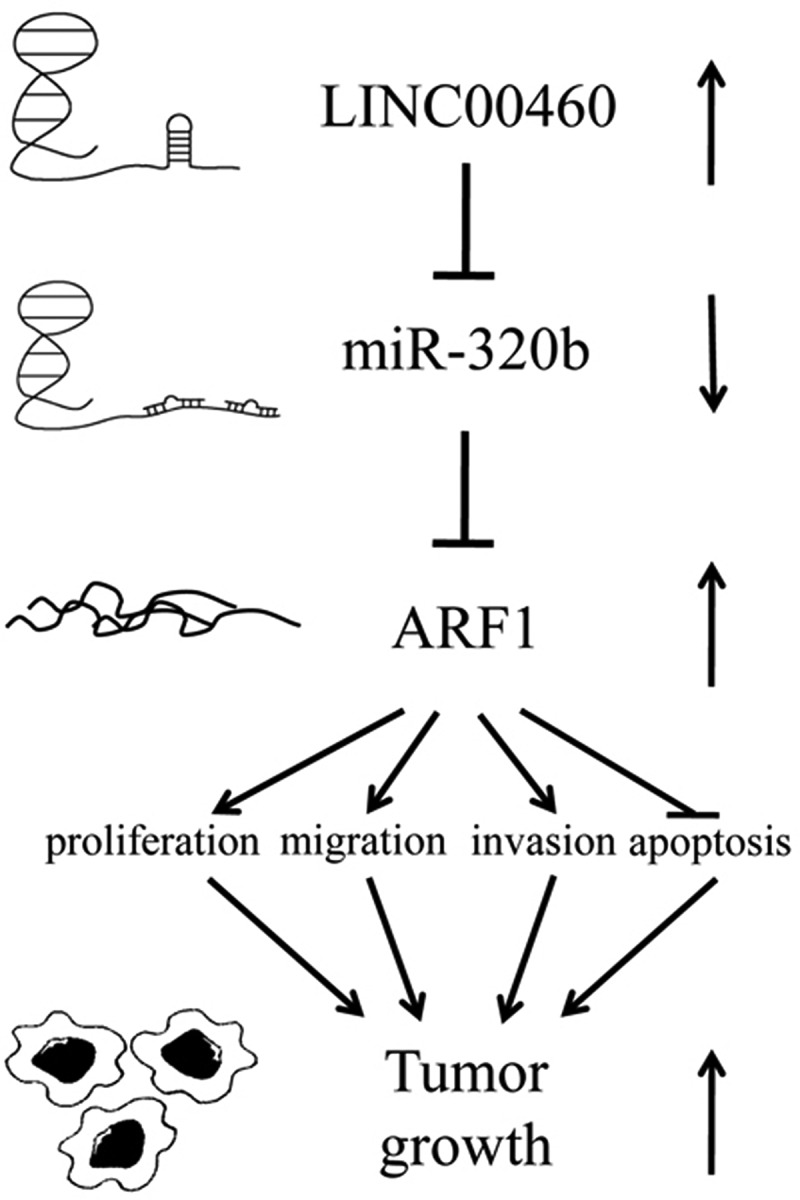

## Introduction

wct Pancreatic adenocarcinoma (PAAD) ranks among the most lethal cancers worldwide with a barely 6% 5-year survival rate [1], and is recognized as a major cause of cancer-related deaths for early metastasis, extensive invasion and poor prognosis [2]. PAAD is commonly diagnosed as distal metastases or at the advanced stage, due to the fact that there are no obvious symptoms in the early stage [3]. Unfortunately, little progress has been made in diagnostic methods and therapeutic strategies, thus the clinical outcome of PAAD patients remains insufficient [4]. Therefore, it is well worth attracting more attention to the understanding of the pathogenesis of PAAD progression, in order to explore effective biomarkers for early diagnosis and therapeutic strategy.

Long non-coding RNA (lncRNAs) is a type of non-coding RNA with a length of more than 200 nucleotides, which potentially acts on gene expression, transcriptional regulation, translation and protein modification [5,6]. The function of lncRNAs is implicated in multiple pathological conditions, especially cancer [7]. Significantly increased LINC00460 level was reported in pancreatic cancer (PC) patients as compared to the healthy controls, indicating that LINC00460 has the potential to be used as an effective biomarker of prognosis and diagnosis of PC [8]. Additionally, previous study also demonstrated marked increase of LINC00460 level in gastric cancer tissues and cell lines and the promoting effect of LINC00460 on the proliferation, migration and invasion of gastric cancer cells, which can be partly reversed by LINC00460 downregulation [9]. Likewise, Xie et al. found overexpressed LINC00460 in head and neck squamous cell carcinoma tissues and cell lines, which promoted cell proliferation, invasion and migration, whereas LINC00460 knockdown abolished this process [10]. It is also found that LINC00460 overexpression promotes hepatocellular carcinoma progression [11]. Interestingly, LINC00460 downregulation could depress PC cell proliferation and promote cell apoptosis, mediated by PI3K/AKT pathway [12]. Moreover, LINC00460 may be a promising biomarker for diagnosis and prognosis of PC, and predicts poor survival of PC patients [13]. However, the underlying mechanism involved in LINC00460 playing its role in the proliferation, migration and invasion of pancreatic cancer cells has been uninvestigated.

We aimed to confirm in this study the role of LINC00460 in PAAD progression and investigate its underlying mechanism in depth. Our findings revealed that LINC00460 boosts the proliferation, migration and invasion of PC cells and hence may represent an effective biomarker for PAAD prognosis and diagnosis.

## Materials and methods

### Cell culture and transfection

Human pancreatic duct cell line H6C7 and pancreatic cancer cell lines (SW1990, HPAC, PaCa-2, CFPAC-1, CAPAN-1) were purchased from American Type Culture Collection (ATCC, Manassas, VA, USA). The cells were cultured in a humidified incubator containing 5% CO_2_ at 37°C, with RPMI-1640 medium (Gibco; Thermo Fisher Scientific, Inc., Waltham, MA, USA) supplemented with 10% fetal bovine serum (FBS, Gibco), 5 mM L-glutamine, 5 mM non-essential amino acid and 100 U/ml penicillin-streptomycin (Invitrogen; Thermo Fisher Scientific, Inc.).

Small interfering RNA (siRNA) against LINC00460 (si-LINC00460), siRNA negative control (si-NC), miR-320b inhibitors and inhibitor-NC were purchased from GenePharma (Shanghai, China). The si-LINC00460 (100 nM) or si-NC, miR-320b inhibitors (1 μM) or inhibitor-NC were transfected into the SW1990 cells using lipofectamine 3000 reagent (Invitrogen, Carlsbad, CA) in accordance with the product instructions. After incubation for 48 h, the SW1990 cells were prepared for further experiments. The success of the transfection was examined by performing qRT-PCR.

### RNA extraction and quantitative real-time PCR (qRT-PCR) analysis

The separation of total RNA was isolated from SW1990 cells was done with a Trizol extraction kit (Life Technologies, USA) following the manufacturer’s protocol. And the reverse-transcription of it into complementary DNA (cDNA) was done with a Prime-Script RT reagent kit (Takara Bio, Inc., Otsu, Japan). Purified mRNA and miRNAs were detected by qRT-PCR assay in combination with SYBR-green real-time Master Mix (Toyobo, Co., Ltd, Osaka, Japan) on an ABI Prism 7300 Sequence Detection system. As an internal control, GAPDH and U6 were measured for normalization and quantification of the expressions of LINC00460 and ARF1, or miR-320b expression, respectively. The 2^−ΔΔCt^ method was applied to calculate the relative expression of target gene.

### CCK8

CCK-8 cell proliferation test kit (Dojindo Molecular Technologies, Tokyo, Japan), was employed for cell viability estimation under the guidance of the product instructions. Transfected SW1990 cells were shortly seeded into 96-well plates with 5 × 10^3^cells per well. CCK8 reagent (10 μl) was added into each well at indicated time points (24, 48 and 72 h), followed by cell incubation for 4 h at 37°C. The absorbance at 450 nm was tested by a microplate reader. Each experiment was conducted in triplicate.

### Colony formation assay

The colony formation assay was performed for the analysis of the biological effect of LINC00460 on cancer cell survival. SW1990 cells were seeded in six-well plates and transfected with or without si-LINC00460. After 14-day incubation, these plates were washed, and the cells were fixed by 4% paraformaldehyde for 15 min, and then stained with 0.1% crystal violet solution before being photographed and counted.

### Wound-healing assay

SW1990 cells (1.5 × 10^5^ cells/well) were plated into six-well plates, and were transfected with or without si-LINC00460 and miR-320b inhibitor at the point of 80–90% confluence. A sterile pipette tip was used to gently induce the wounds on the monolayered cells, which were then incubated in 5% CO_2_ at 37°C for 24 h. At last, the wounds were observed and photographed under a microscope (Nikon).

### Transwell chamber assay

After transfection, SW1990 cells were first stored in the medium free of serum, followed by addition of cell suspension (2 × 10^4^ cells/well) to the upper chamber (Corning, New York, USA) precoated with Membrane Matrix (BD Biosciences, San Jose, CA, USA), and addition of 0.5 mL medium with 10% FBS to the lower chamber. After incubation for 24 h at 37°C in 5% CO_2_ under humidification, the cells remaining on the upper surface were wiped off with a cotton swab. The cells on the lower surface of the membrane were fixed with methanol and stained with 1% crystal violet for 10 min. Cells that penetrated the membrane were counted microscopically from five random visual angles (200× magnification).

### Flow cytometry

After transfection, SW1990 cells were harvested for flow cytometry. Shortly after double staining by Annexin V-FITC/PI apoptosis kit (Thermo Fisher Scientific) following the manufacturer’s protocols, SW1990 cell apoptosis was examined by a flow cytometer (BD, NJ, USA). The above experiment was replicated three times.

### Western blot

RIPA lysis buffer was utilized to extract the total protein samples of SW1990 cells, and the concentration of which was quantified with the use of the BCA assay kits (Beyotime, China). An equal number of proteins were fractionated with 12% SDS-PAGE and transferred to PVDF membranes. Primary antibodies against the following proteins were used: Bcl-2, Bax, caspase3, pro-caspase3, caspase 7, pro-caspase 7, ARF1. The density of protein bands was analyzed by ImageJ software.1.48 and normalized to GAPDH.

### Luciferase reporter assay

Before transfection, 293 T cells (5 × 10^4^ cells/well) were plated in 24-well plates and cultured overnight. First of all, the wildtype (WT) and the mutant sequence of LINC00460 were, respectively, sub-cloned into luciferase reporter vectors (PsiCHECK2, Promega, USA). Then the cells were co-transfected with PsiCHECK2 vectors and miR-320b mimic or vector. After incubation for 48 h, the fireﬂy luciferase activity was measured and normalized based on fireﬂy and Renilla luciferases using the Dual-Luciferase Reporter Assay System (Promega, Madison, WI, USA). For confirmation of miR-320b/ARF1 binding, the WT 3ʹ-UTR sequence of ARF1 containing miR-320b binding sites was sub-cloned into PsiCHECK2 luciferase reporter vector, referring to the protocols of similar co-transfection and fluorescence measurement. The above plasmids and reporter gene carriers were all synthesized by GenePharma (Shanghai, China).

### Statistical analysis

Statistical data were analyzed by GraphPad Prism 8.0. All data were expressed as the means ± standard error (SEM). One-way ANOVA followed Tukey’s test were successively performed to analyze the differences among the means. Differences at *P* < 0.05 were considered statistically significant.

## Results

### LINC00460 knockdown inhibited the viability and proliferation of SW1990 cells

**Figure 1.**
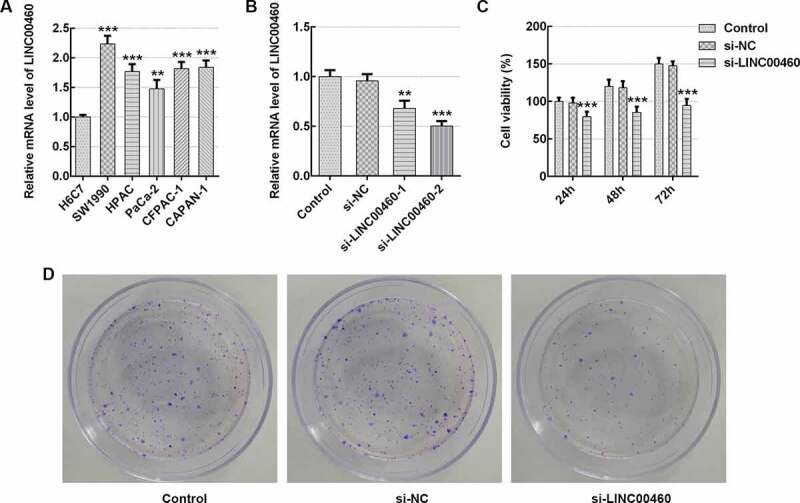
LINC00460 knockdown inhibited the cell growth of SW1990 cells. (a–b) The LINC00460 mRNA expression was quantified by qRT-PCR. (c) The cell viability of SW1990 cells was analyzed by CCK8 assay. (d) The cell proliferation of SW1990 cells was analyzed by colony formation assay. Error bars represent the mean ± SEM from three independent experiments. **P< 0.01, ***P< 0.001 *vs*. Control

To explore the role of LINC00460 in PAAD progression, the LINC00460 mRNA level was detected by qRT-PCR in multiple pancreatic cancer cell lines. Figure 1(a) exhibits a significant increase in the level of LINC00460 mRNA in multiple pancreatic cancer cell lines by comparison with the normal pancreatic duct cell line H6C7, with it being most noticeable in SW1990 cells. Hence, the SW1990 cell line was selected in subsequent experimentation. Subsequently, the si-LINC00460-1 (5ʹ-CCATCCACTTCAAAGTATT-3ʹ) and si-LINC00460-2 (5ʹ-CCTCTGAAATGGTGACAAT-3ʹ) were conducted by Shanghai GenePharma, Co., Ltd. to achieve the LINC00460 downregulation. The transfection efficiency was determined by qRT-PCR. As shown in Figure 1(b), the LINC00460 mRNA level in si-LINC00460-2 group is lower than that in si-LINC00460-1 group. Hence, si-LINC00460-2 was chosen for further experimentations. The CCK8 results demonstrated that LINC00460 downregulation significantly depressed the viability of SW1990 cells at the indicated time (24, 48, 72 h), especially at 48 and 72 h (Figure 1(c)). The results of colony formation assay presented that LINC00460 knockdown remarkably decreased SW1990 cell proliferation in contrast to the control (Figure 1(d)). These results suggested an inhibitory effect of LINC00460 knockdown on SW1990 cell growth.

### LINC00460 knockdown inhibited the migration and invasion of SW1990 cells

**Figure 2.**
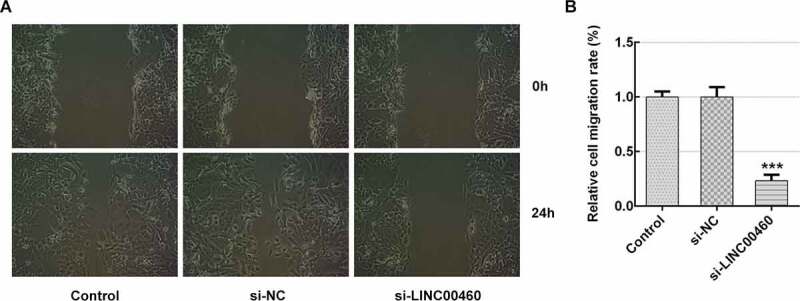
LINC00460 knockdown inhibited the migration of SW1990 cells. (a–b) The migration of SW1990 cells transfected with si-LINC00460 or si-NC was detected by wound-healing assay. Error bars represent the mean ± SEM from three independent experiments. ***P< 0.001 *vs*. Control

**Figure 3.**
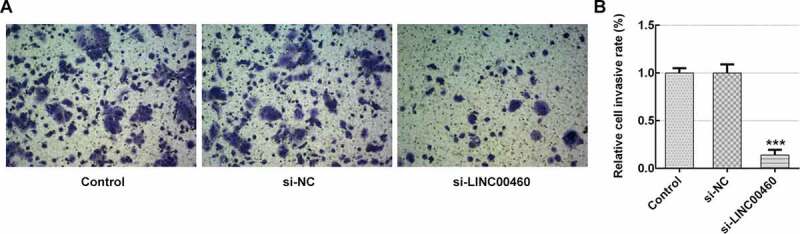
LINC00460 knockdown inhibited the invasion of SW1990 cells. (a–b) The invasion of SW1990 cells transfected with si-LINC00460 or si-NC was detected by transwell chamber assay. Error bars represent the mean ± SEM from three independent experiments. ***P< 0.001 *vs*. Control

To further verify the role of LINC00460 in PAAD progression, the migration and invasion of SW1990 cells were detected by wound-healing assay and transwell chamber assay, respectively. Figure 2 demonstrates that LINC00460 downregulation led to a great reduction in migratory capability compared to the control. Furthermore, the invasive capability in si-LINC00460 was lower than that in the control group (Figure 3). These results implied that LINC00460 knockdown might prevent the migration and invasion of SW1990 cells.

### LINC00460 knockdown promoted the apoptosis of SW1990 cells

**Figure 4.**
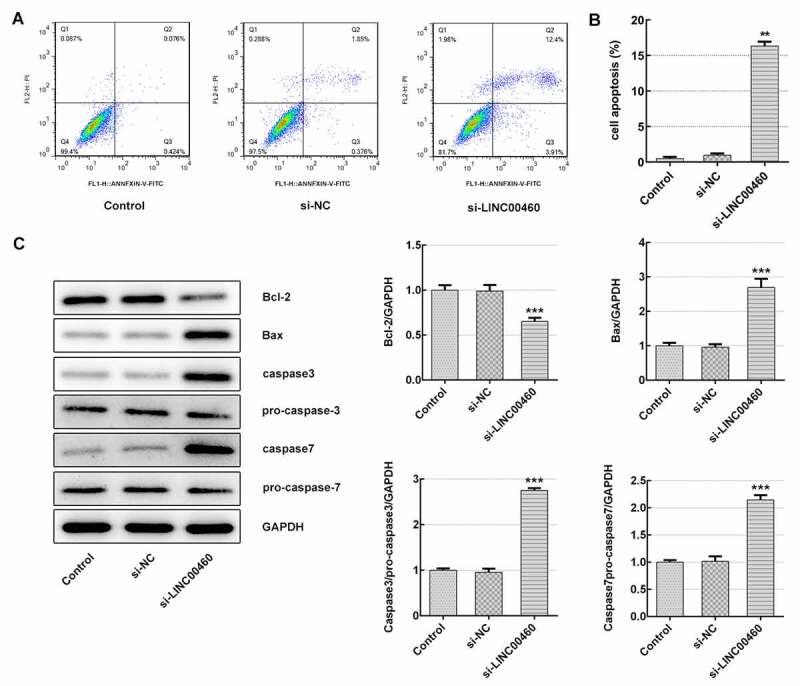
LINC00460 knockdown promoted the apoptosis of SW1990 cells. (a–b) The apoptosis rate of SW1990 cells was detected by flow cytometry. (c) The expressions of proteins including Bcl-2, Bax, caspase3, pro-caspase3, caspase7 and pro-caspase7 were determined by western blotting. Error bars represent the mean ± SEM from three independent experiments. **P< 0.01, ***P< 0.001 *vs*. Control

Subsequently, the apoptosis rate was detected to further clarify how LINC00460 acts on cell growth. The results of flow cytometry identified that LINC00460 knockdown largely improved the apoptosis rate of SW1990 cells (Figure 4(a-b)). Furthermore, the expression of proteins related to cell apoptosis was determined with western blotting. Figure 4(c) demonstrates that the Bcl-2 (anti-apoptosis) expression was decreased, and the expression levels of Bax (pro-apoptosis), caspase3 and caspase7 were significantly increased in SW1990 cells of si-LINC00460 compared to the control group, suggesting that LINC00460 knockdown promoted the apoptosis of SW1990 cells.

### LINC00460 directly targeted miR-320b and downregulated miR-320b expression

**Figure 5.**
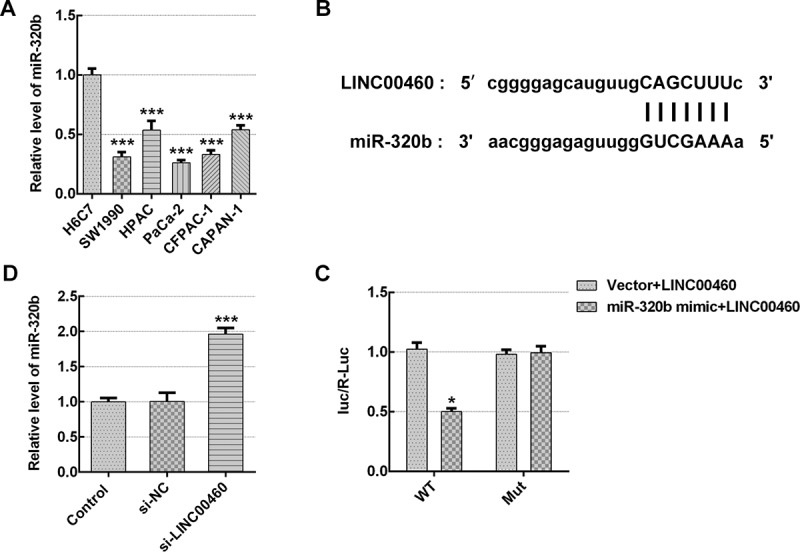
LINC00460 directly target miR-320b and downregualted miR-320b expression. (a) The mRNA expression of miR-320b in multiple pancreatic cancer cell lines was quantified with qRT-PCR. (b) Binding sites of LINC00460 and miR-320b predicted by Starbase website. (c) The relative luciferase activity was detected by luciferase reporter assay. (d) The mRNA expression of miR-320b in SW1990 cells transfected with si-LINC00460 or si-NC was quantified with qRT-PCR. Error bars represent the mean ± SEM from three independent experiments. *P< 0.05, ***P< 0.001 *vs*. Control

To investigate the potential mechanism involved in the role of LINC00460 in PAAD progression, Starbase database (http://starbase.sysu.edu.cn/) was consulted for the prediction of LINC00460 and miR-320b interacting with each other, as shown in Figure 5(b). Firstly, the miR-320b mRNA level was detected in H6C7 cells and pancreatic cancer cell lines (SW1990, HPAC, PaCa-2, CFPAC-1, CAPAN-1). The qRT-PCR results established lower miR-320b mRNA level in pancreatic cancer cells than H6C7 cells, especially in SW1990 and PaCa-2 cell lines (Figure 5(a)). Subsequently, we performed the luciferase reporter assay for the purpose of confirming the direct binding ability of LINC00460/miR-320b. As shown in Figure 5(c), co-transfection of miR-320b mimic noticeably dampened the relative luciferase activity of LINC00460-WT without changing mutant vector activity, suggesting that LINC00460 can directly target miR-320b. Interestingly, the qRT-PCR suggested that LINC00460 knockdown led to significant miR-320b upregulation (Figure 5(d)), which suggested that LINC00460 directly targeted miR-320b and upregulated miR-320b expression.

### MiR-320b downregulation partly reversed the effect of LINC00460 knockdown on the proliferation, migration, invasion and apoptosis of SW1990 cells

**Figure 6.**
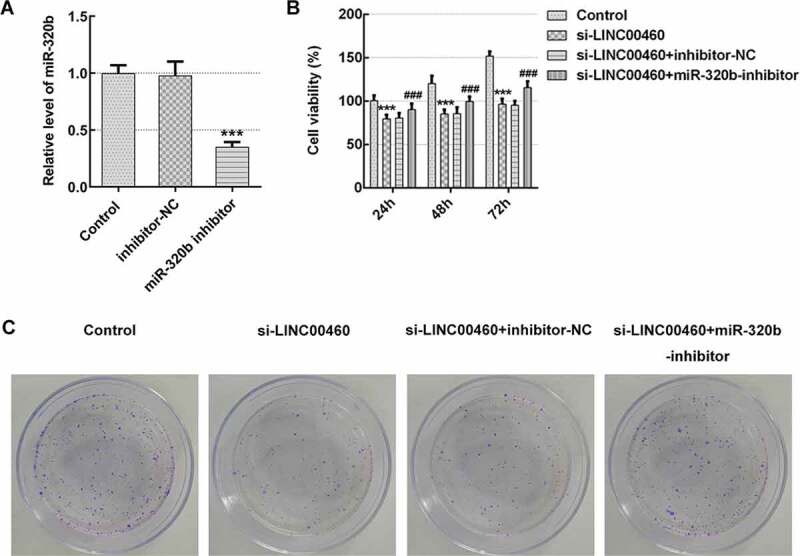
MiR-320b dowregulation partly reversed the effect of LINC00460 knockdown on the proliferation of SW1990 cells. (a) The mRNA expression of miR-320b in SW1990 cells transfected with miR-320b inhibitor or inhibitor-NC was quantified with qRT-PCR. (b) The cell viability of SW1990 cells was analyzed by CCK8 assay. (d) The cell proliferation of SW1990 cells was analyzed by colony formation assay. Error bars represent the mean ± SEM from three independent experiments. ***P< 0.001 *vs*. Control. ^###^*P*< 0.001 *vs*. si-LINC00460

**Figure 7.**
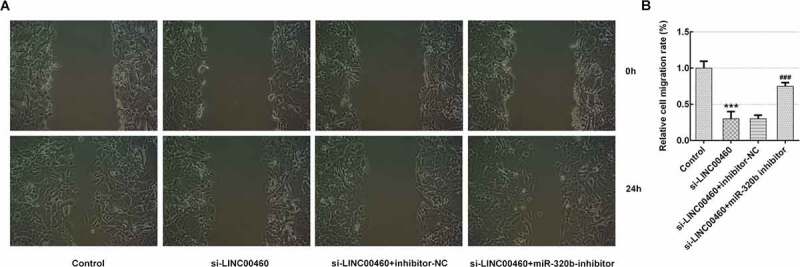
MiR-320b dowregulation partly reversed the effect of LINC00460 knockdown on the migration of SW1990 cells. (a–b) The migration of SW1990 cells transfected with si-LINC00460, si-LINC00460+ inhibitor-NC or si-LINC00460+ miR-320b-inhibitor was detected by wound-healing assay. Error bars represent the mean ± SEM from three independent experiments. ***P< 0.001 *vs*. Control. ^###^*P*< 0.001 *vs*. si-LINC00460

**Figure 8.**
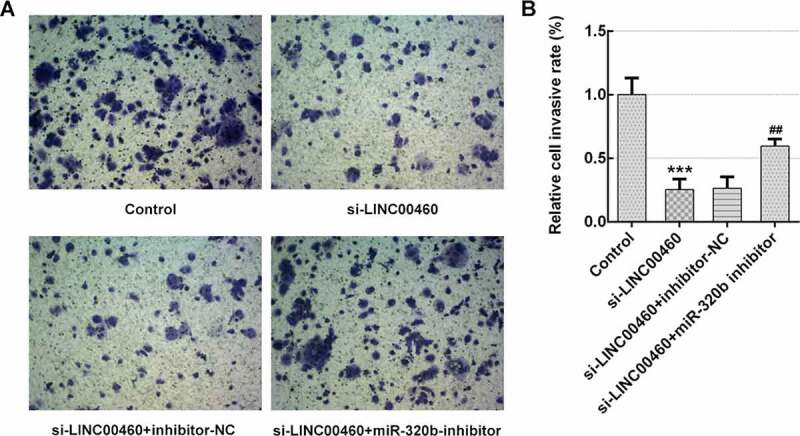
MiR-320b dowregulation partly reversed the effect of LINC00460 knockdown on the invasion of SW1990 cells. (a–b) The invasion of SW1990 cells transfected with si-LINC00460, si-LINC00460+ inhibitor-NC or si-LINC00460+ miR-320b-inhibitor was detected by transwell chamber assay. Error bars represent the mean ± SEM from three independent experiments. ***P< 0.001 *vs*. Control. ^##^*P*< 0.01 *vs*. si-LINC00460

**Figure 9.**
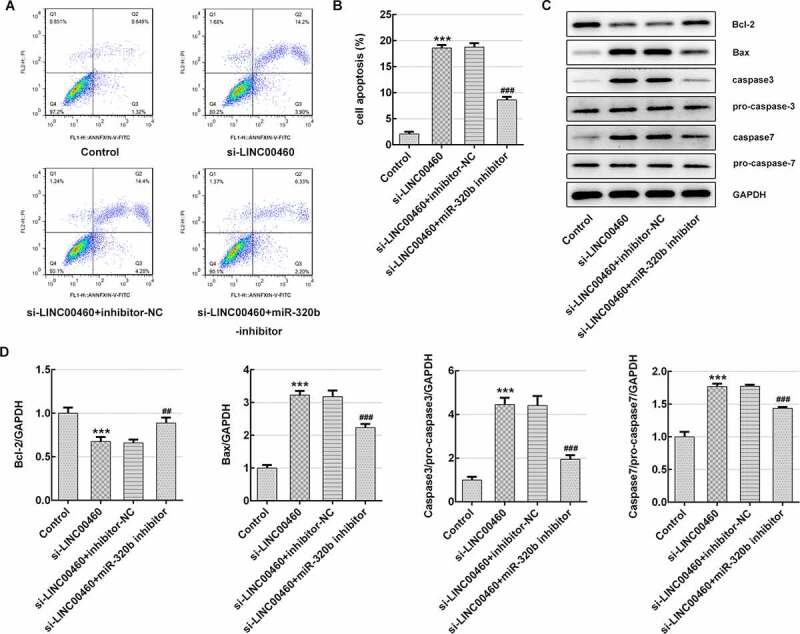
MiR-320b dowregulation partly reversed the effect of LINC00460 knockdown on the apoptosis of SW1990 cells. (a–b) The apoptosis rate of SW1990 cells was detected by flow cytometry. (c) The expressions of proteins including Bcl-2, Bax, caspase3, pro-caspase3, caspase7 and pro-caspase7 were determined by western blotting, and quantification (d). Error bars represent the mean ± SEM from three independent experiments. ***P< 0.001 *vs*. Control. ^##^*P*< 0.01, ^###^*P*< 0.001 *vs*. si-LINC00460

To corroborate the underlying mechanism of LINC00460 in pancreatic cancer cell growth, the cellular behaviors were further analyzed under the conditions of miR-320b downregulation, which was achieved through transfection of miR-320b inhibitor plasmids (10 nM) as we previously described. The miR-320b inhibitor (5ʹ-UUGCCCUCUCAACCCAGCUUUU-3ʹ) and its negative control (inhibitor-NC, 5ʹ-CAGUACUUUUGUGUAGUACAA-3ʹ) were synthesized by GenePharma (Shanghai, China). The qRT-PCR result revealed that miR-320b mRNA level was notably decreased by miR-320b inhibitor (Figure 6(a)), suggesting that miR-320b inhibitor effectively downregulated the miR-320b expression. CCK8 results suggested that cell viability was significantly enhanced in si-LINC00460+ miR-320b inhibitor group compared to si-LINC00460 group (Figure 6b), as well as the proliferative ability of SW1990 cells (Figure 6c). Moreover, miR-320b inhibition partly reversed the suppressive effect of LINC00460 knockdown on migratory capability of SW1990 cells (Figure 7a-B). It was supported by transwell chamber assay that miR-320b downregulation remarkably elevated the cell invasive rate even under the condition of LINC00460 knockdown (Figure 8(a-b)). Furthermore, lower apoptosis rate of SW1990 cells in si-LINC00460+ miR-320b inhibitor group was observed in comparison to the si-LINC00460 group (Figure 9(a-b)). Additionally, miR-320b inhibition led to Bcl-2 upregulation and reduction in expressions of Bax, caspase3 and caspase7, compared to si-LINC00460 group (Figure 9(c-d)). These results implied that miR-320b downregulation partly reversed the effect of LINC00460 knockdown on the proliferation, migration, invasion and apoptosis of SW1990 cells.

### LINC00460 knockdown inhibited the proliferation, migration and invasion, and promoted apoptosis of SW1990 cells via modulation of the miR-320b/ARF1 axis

**Figure 10.**
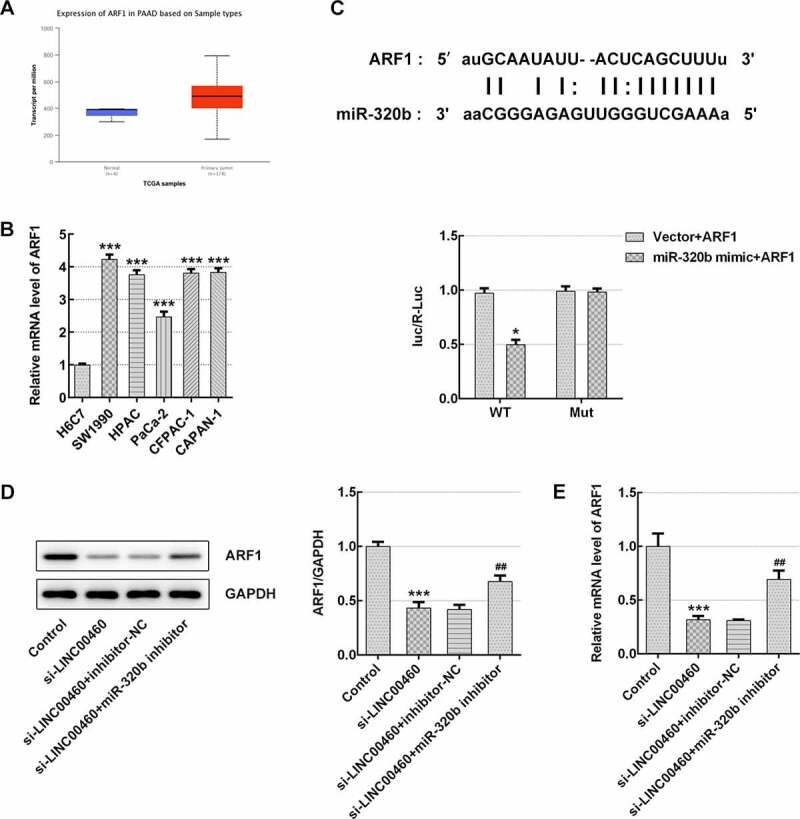
miR-320b/ARF1 axis mediated the effect of LINC00460 knockdown on cell growth of SW1990 cells. (a) The ARF1 level in PAAD patients by UALCAN website. (b) The ARF1 mRNA level was detected by qRT-PCR. (c) Binding sites of miR-320b and ARF1 predicted by Starbase website, and the relative luciferase activity was detected by luciferase reporter assay. (d) The ARF1 proteins expression was quantified by western blotting. (e) The ARF1 mRNA level was detected by qRT-PCR. Error bars represent the mean ± SEM from three independent experiments. *P< 0.05, ***P< 0.001 *vs*. Control; ^##^*P*< 0.01 *vs*. si-LINC00460

It was found that ARF1is highly expressed in PAAD patients by searching on UALCAN website (http://ualcan.path.uab.edu/index.html), as shown in Figure 10(a). The results of qRT-PCR showed relatively elevated mRNA level of ARF1 in pancreatic cancer cell lines (SW1990, HPAC, PaCa-2, CFPAC-1, CAPAN-1), compared with H6C7 cells (Figure 10(b)). A noteworthy fact is that we carried out the luciferase reporter assay based on Starbase prediction of interaction between miR-320b and ARF1, and noticed that co-transfection of miR-320b mimic markedly weakened the relative luciferase activity of ARF1-WT without changing that of the mutant vector, which suggests that ARF1 may be one of the direct targets of miR-320b (Figure 10(c)). Finally, the western blotting and qRT-PCR were performed to detect the protein expression and mRNA level of ARF1. As shown in Figure 10(d-e), INC00460 knockdown significantly inhibited ARF1 expression in the aspect of both mRNAs and proteins, whereas miR-320b inhibition reversed such inhibitory effect (Figure 10(d-e)). These results suggested that miR-320b/ARF1 axis may mediate the function that LINC00460 knockdown performs in the proliferation, migration and invasion, and apoptosis of SW1990 cells.

## Discussion

LncRNAs, acting as an oncogene or tumor suppressor, play a vital regulatory role in the occurrence and the progression of cancers according to previous studies [14,15]. It has been reported that LINC00460, a novel lncRNA, is essential for the onset and the development of multiple cancers such as head and neck squamous cell carcinoma [16], hepatocellular carcinoma [17], lung cancer [18], colorectal cancer [19], etc. However, the role of LINC00460 in PAAD progression has not been well clarified. Hence, the aim of the present study was to investigate the underlying mechanism of LINC00460 in PAAD progression.

Previous study suggested that LINC00460 level exhibits high expression in human prostate cancer tissues and cell lines, and downregulation of LINC00460 suppresses cell proliferation and promotes cell apoptosis in prostate cancer, which is consistent with our study [12]. Moreover, the LINC00460 level is positively related to tumor size, and predicts poor survival of PC patients [13]. Interestingly, our findings indicated that LINC00460 level was increased in pancreatic cancer cell lines (SW1990, HPAC, PaCa-2, CFPAC-1, CAPAN-1) compared to normal human pancreatic duct cells. Moreover, LINC00460 knockdown inhibited the proliferation, migration and invasion, as well as promoting the apoptosis of pancreatic cancer cells. Thus, LINC00460 expression level may be closely related to PAAD pathogenesis. Here, we described how LINC00460 works on PAAD progression, namely its mechanism, through a sequence of investigations.

To explore the biochemical mechanism related to LINC00460 functioning in PAAD progression, the potential targets of LINC00460 were predicted on Starbase website. Fortunately, it is found that LINC00460 can directly target miR-320b and that LINC00460 downregulation significantly increased the miR-320b level. Notably, miR320 is reported to have suppressive effect on proliferation, migration and invasion in carcinomas [20,21]. Recent study demonstrated that miR-320b downregulation contributed to the rapid reproduction, invasion and epithelial–mesenchymal transition of pancreatic cancer cells [22]. Additionally, it was reported that the miR-320b expression in pancreatic cancer cells was lower than the normal pancreatic cells, and miR-320b inhibited the proliferation of pancreatic cancer cells [23]. Here, our findings revealed the restorative effect of miR-320b on cell proliferation, migration, invasion and apoptosis triggered by LINC00460 knockdown, suggesting that LINC00460/miR-320b axis may be essential elements for PAAD progression.

Interestingly, we further found that miR-320b can directly bind ARF1 by the prediction on Starbase website. Simultaneously, the searched data of UALCAN website revealed that ARF1 is highly expressed in PAAD patients. In our study, qRT-PCR results implied much increased mRNA expression of ARF1 in pancreatic cancer cell lines (SW1990, HPAC, PaCa-2, CFPAC-1, CAPAN-1), compared with H6C7 cells. Jason E *et al*. demonstrated that ARF1 activation promoted cell proliferation in prostate cancer progression [24]. Additionally, ARF1 expression level is related to the grade of ovarian cancer, and its overexpression proliferation and migration of ovarian cancer cells [25]. The current study suggested that ARF1 expression regulated by lncRNA TP73-AS1 exerts regulative effect on cervical cell proliferation and migration [26]. Moreover, Liwei *et al*. have reported that blockade of ARF1 activation is a promising therapeutic strategy for PAAD treatment [27]. Intriguingly, our results suggested that INC00460 knockdown significantly inhibited ARF1 expression, whereas miR-320b downregulation could reverse such effect. Taken together, the above findings supported the inhibitory effect of LINC00460 knockdown on cell proliferation, migration and invasion and the promoting effect of it on the apoptosis of SW1990 cells, both via modulation of miR-320b/ARF1 axis.

## Conclusion

In summary, our study elucidated that downregulation of LINC00460 inhibited the proliferation, migration and invasion, and promoted the apoptosis of pancreatic cancer cells via modulation of miR-320b/ARF1 axis. Hence, targeting LINC00460 may present a novel promising therapeutic strategy for PAAD treatment.

## Supplementary Material

Supplemental Material

## Article Highlights

- LINC00460 knockdown inhibited the metastasis of pancreatic cancer cells.
- LINC00460 directly target miR-320b and downregualted miR-320b expression.
- LINC00460 knockdown led to a reduction in ARF1 expression.

## References

[1] Siegel RL, Miller KD, Jemal A. Cancer statistics, 2018. CA Cancer J Clin. 2018;68(1):7–30. 29313949 10.3322/caac.21442

[2] Ando Y, Ohuchida K, Otsubo Y, et al. Necroptosis in pancreatic cancer promotes cancer cell migration and invasion by release of CXCL5. PLoS One. 2020;15(1):e0228015. 31999765 10.1371/journal.pone.0228015 PMC6991976

[3] Garcia-Carbonero N, Li W, Cabeza-Morales M, et al. New hope for pancreatic ductal adenocarcinoma treatment targeting endoplasmic reticulum stress response: a systematic review. Int J Mol Sci. 2018;19(9):2468. 10.3390/ijms19092468 PMC6165247 30134550

[4] McGuigan A, Kelly P, Turkington RC, et al. Pancreatic cancer: a review of clinical diagnosis, epidemiology, treatment and outcomes. World J Gastroenterol. 2018;24(43):4846–4861. 30487695 10.3748/wjg.v24.i43.4846 PMC6250924

[5] Huang X, Xiao R, Pan S, et al. Uncovering the roles of long non-coding RNAs in cancer stem cells. J Hematol Oncol. 2017;10(1):62. 28245841 10.1186/s13045-017-0428-9 PMC5331729

[6] Wawrzyniak O, Zarﺅﻷbska ﻊ, Rolle K, et al. Circular and long non-coding RNAs and their role in ophthalmologic diseases. Acta Biochim Pol. 2018;65(4):497–508. 30428483 10.18388/abp.2018_2639

[7] Chan JJ, Tay Y. Noncoding RNA: RNA regulatory networks in cancer. Int J Mol Sci. 2018;19(5):1310. 10.3390/ijms19051310 PMC5983611 29702599

[8] Wang Y, Li Z, Zheng S, et al. Expression profile of long non-coding RNAs in pancreatic cancer and their clinical significance as biomarkers. Oncotarget. 2015;6(34):35684–35698. 26447755 10.18632/oncotarget.5533 PMC4742134

[9] Wang F, Liang S, Liu X, et al. LINC00460 modulates KDM2A to promote cell proliferation and migration by targeting miR-342-3p in gastric cancer. Onco Targets Ther. 2018;11:6383–6394. 30323616 10.2147/OTT.S169307 PMC6174301

[10] Xie X, Xiong G, Wang Q, et al. Long non-coding RNA LINC00460 promotes head and neck squamous cell carcinoma cell progression by sponging miR-612 to up-regulate AKT2. Am J Transl Res. 2019;11(10):6326–6340. 31737186 PMC6834525

[11] Jin J, Xu H, Li W, et al. LINC00346 acts as a competing endogenous RNA regulating development of hepatocellular carcinoma via modulating CDK1/CCNB1 axis. Front Bioeng Biotechnol. 2020;8:54. 32133348 10.3389/fbioe.2020.00054 PMC7039823

[12] Dong Y, Quan HY. Downregulated LINC00460 inhibits cell proliferation and promotes cell apoptosis in prostate cancer. Eur Rev Med Pharmacol Sci. 2019;23(14):6070–6078. 31364108 10.26355/eurrev_201907_18420

[13] Sun J, Yang J, Lv K, et al. Long non-coding RNA LINC00460 predicts poor survival and promotes cell viability in pancreatic cancer. Oncol Lett. 2020;20(2):1369–1375. 32724379 10.3892/ol.2020.11652 PMC7377077

[14] Cai C, Yang L, Tang Y, et al. Prediction of overall survival in gastric cancer using a nine-lncRNA. DNA Cell Biol. 2019;38(9):1005–1012. 31335180 10.1089/dna.2019.4832

[15] Zhuang C, Ma Q, Ye J, et al. LncRNA GClnc1 promotes proliferation and invasion of bladder cancer through activation of MYC. Faseb J. 2019;33(10):11045–11059. 31298933 10.1096/fj.201900078RR

[16] Jiang Y, Cao W, Wu K, et al. LncRNA LINC00460 promotes EMT in head and neck squamous cell carcinoma by facilitating peroxiredoxin-1 into the nucleus. J Exp Clin Cancer Res. 2019;38(1):365. 31429766 10.1186/s13046-019-1364-z PMC6700841

[17] Tu J, Zhao Z, Xu M, et al. LINC00460 promotes hepatocellular carcinoma development through sponging miR-485-5p to up-regulate PAK1. Biomed Pharmacother. 2019;118:109213. 31376654 10.1016/j.biopha.2019.109213

[18] Li K, Sun D, Gou Q, et al. Long non-coding RNA linc00460 promotes epithelial-mesenchymal transition and cell migration in lung cancer cells. Cancer Lett. 2018;420:80–90. 29409808 10.1016/j.canlet.2018.01.060

[19] Lian Y, Yan C, Xu H, et al. A novel lncRNA, LINC00460, affects cell proliferation and apoptosis by regulating KLF2 and CUL4A expression in colorectal cancer. Mol Ther Nucleic Acids. 2018;12:684–697. 30092404 10.1016/j.omtn.2018.06.012 PMC6083012

[20] Li Y, Tang X, He Q, et al. Overexpression of mitochondria mediator gene TRIAP1 by miR-320b loss is associated with progression in nasopharyngeal carcinoma. PLoS Genet. 2016;12(7):e1006183. 27428374 10.1371/journal.pgen.1006183 PMC4948882

[21] Lv GY, Miao J, Zhang XL. Long noncoding RNA XIST promotes osteosarcoma progression by targeting Ras-related protein RAP2B via miR-320b. Oncol Res. 2018;26(6):837–846. 28409547 10.3727/096504017X14920318811721 PMC7844768

[22] Cao W, Zhou G. LncRNA SNHG12 contributes proliferation, invasion and epithelial-mesenchymal transition of pancreatic cancer cells by absorbing miRNA-320b. Biosci Rep. 2020;40(6). DOI:10.1042/bsr20200805 PMC7276652 32432698

[23] Jingyang Z, Jinhui C, Lu X, et al. Mir-320b inhibits pancreatic cancer cell proliferation by targeting FOXM1. Curr Pharm Biotechnol. 2020. DOI:10.2174/1389201021999200917144704 32942974

[24] Davis JE, Xie X, Guo J, et al. ARF1 promotes prostate tumorigenesis via targeting oncogenic MAPK signaling. Oncotarget. 2016;7(26):39834–39845. 27213581 10.18632/oncotarget.9405 PMC5129974

[25] Gu G, Chen Y, Duan C, et al. Overexpression of ARF1 is associated with cell proliferation and migration through PI3K signal pathway in ovarian cancer. Oncol Rep. 2017;37(3):1511–1520. 28098897 10.3892/or.2017.5388

[26] Xu J, Zhang J. LncRNA TP73-AS1 is a novel regulator in cervical cancer via miR-329-3p/ARF1 axis. J Cell Biochem. 2020;121(1):344–352. 31232491 10.1002/jcb.29181

[27] Lang L, Shay C, Zhao X, et al. Combined targeting of Arf1 and Ras potentiates anticancer activity for prostate cancer therapeutics. J Exp Clin Cancer Res. 2017;36(1):112. 28830537 10.1186/s13046-017-0583-4 PMC5568197

