# Supplementary material for: Downregulation of long non-coding RNA LINC00460 inhibits the proliferation, migration and invasion, and promotes apoptosis of pancreatic cancer cells via modulation of the miR-320b/ARF1 axis
Source: Bioengineered. 2020 Dec 21;12(1):96–107. doi: 10.1080/21655979.2020.1863035 (PMC8806231; doi:10.1080/21655979.2020.1863035)
Supplement: Supplemental Material [file KBIE_A_1863035_SM0156.zip › supplement/Primer sequence.docx]

The sequences of the primers used in RT-qPCR were as follows:

LINC00460 forward 5ʹ-GTGGATGAGAACGAAGGTTACG-3ʹ, reverse 5ʹ-CTTTCCCACGCTCAGTCTTT-3ʹ;

ARF1 forward 5ʹ-GGGGAGGCAAACCGGTCA-3ʹ, reverse 5ʹ-GGCTTCTAAACCTAGTGCCTGG-3ʹ;

miR-320b forward 5ʹ-GATGCTGAAAAGCTGGGTTG-3ʹ, 5ʹ-TATGGTTGTTCTGCTCTCTGTCTC-3ʹ;

U6 forward 5ʹ-CTCGCTTCGGCAGCACA-3ʹ, reverse 5ʹ-AACGCTTCACGAATTTGCGT-3ʹ;

GAPDH forward 5ʹ-TCGGAGTCAACGGATTTGGT-3ʹ, reverse 5ʹ-TTGGAGGGATCTCGCTCCT-3ʹ.
