# Supplementary figures and images for: Downregulation of long non-coding RNA LINC00460 inhibits the proliferation, migration and invasion, and promotes apoptosis of pancreatic cancer cells via modulation of the miR-320b/ARF1 axis
Source: Bioengineered. 2020 Dec 21;12(1):96–107. doi: 10.1080/21655979.2020.1863035 (PMC8806231; doi:10.1080/21655979.2020.1863035)

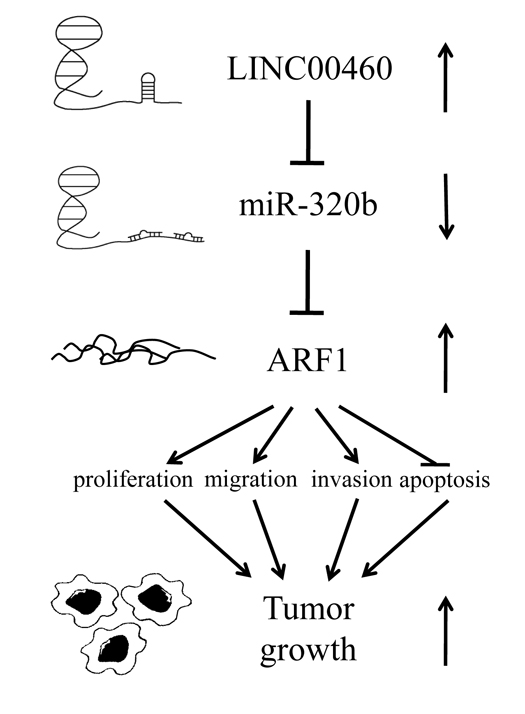

Supplement: Supplemental Material [file KBIE_A_1863035_SM0156.zip › supplement/GraphicalAbstract.jpg]

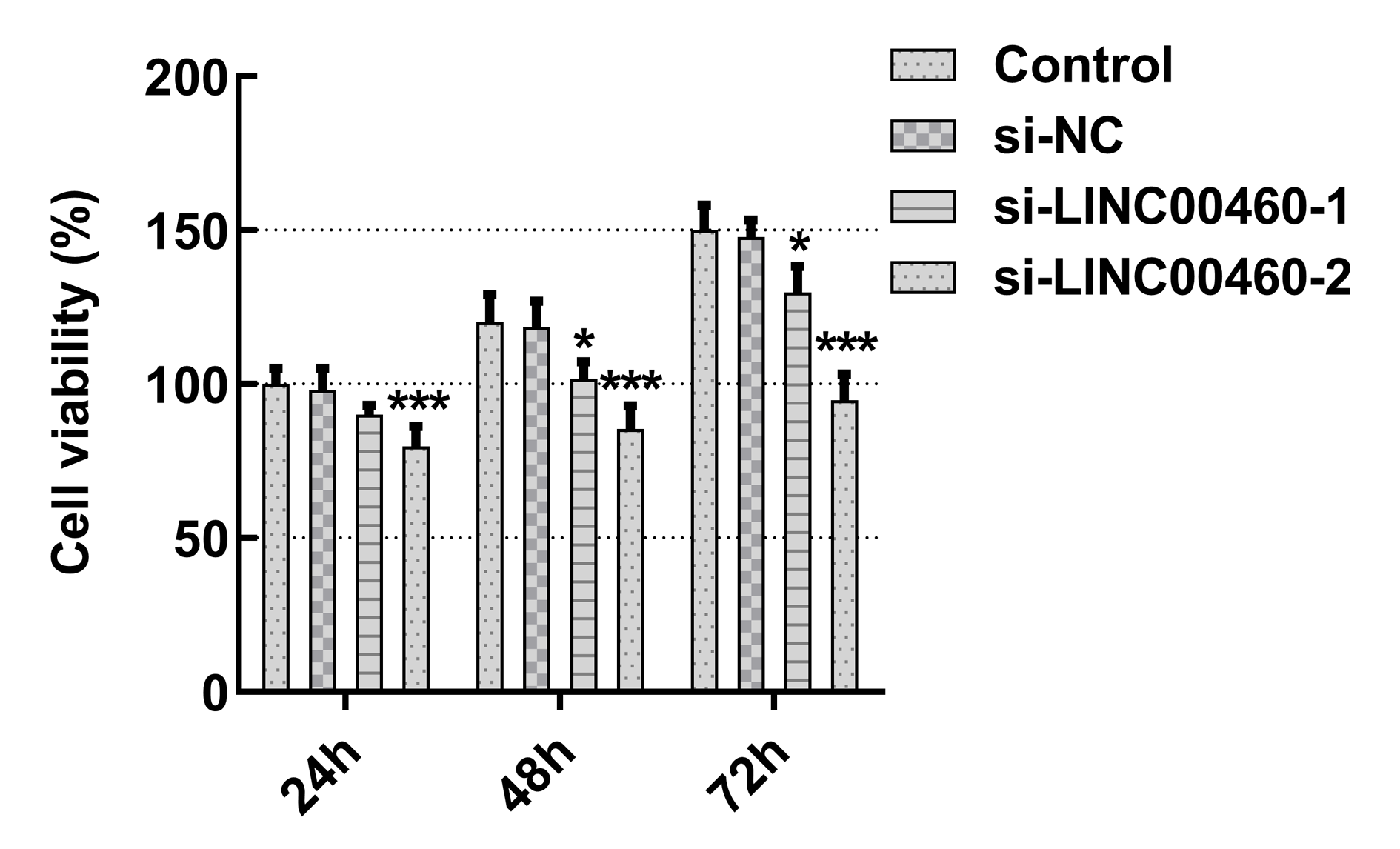

Supplement: Supplemental Material [file KBIE_A_1863035_SM0156.zip › supplement/Supplementary file CCK8.tif]
